# Establishment of a biomarker model for predicting bone metastasis in resected stage III non-small cell lung cancer

**DOI:** 10.1186/1756-9966-31-34

**Published:** 2012-04-26

**Authors:** Zhen Zhou, Zhi-Wei Chen, Xiao-Hua Yang, Lan Shen, Xing-Hao Ai, Shun Lu, Qing-Quan Luo

**Affiliations:** 1 Department of Lung Tumor Clinical Medical Center, Shanghai Chest Hospital affiliated to Shanghai Jiaotong University, and Thoracic Tumor Clinical Medicine Center of Shanghai Municipality, Shanghai, 200032, China; 2 Tumor Research Lab, Shanghai Chest Hospital affiliated to Shanghai Jiaotong University, Shanghai, 200030, China; 3 Department of Lung Tumor Clinical Medical Center, Shanghai Chest Hospital affiliated to Shanghai Jiaotong University, and Thoracic Tumor Clinical Medicine Center of Shanghai Municipality, No 241, West Huaihai Road, Xuhui District, Shanghai, 200032, China

**Keywords:** Lung cancer, Bone metastasis, Biomarker

## Abstract

**Background:**

This study was designed to establish a biomarker risk model for predicting bone metastasis in stage III non-small cell lung cancer (NSCLC).

**Methods:**

The model consists of 105 cases of stage III NSCLC, who were treated and followed up. The patients were divided into bone metastasis group (n = 45) and non-bone metastasis group (other visceral metastasis and those without recurrence) (n = 60). Tissue microarrays were constructed for immunohistochemical study of 10 molecular markers associated with bone metastasis, based on which a model was established via logistic regression analysis for predicting the risk of bone metastases. The model was prospectively validated in another 40 patients with stage III NSCLC.

**Results:**

The molecular model for predicting bone metastasis was logit (P) = − 2.538 + 2.808 CXCR4 +1.629 BSP +0.846 OPN-2.939 BMP4. ROC test showed that when P ≥ 0.408, the sensitivity was up to 71% and specificity of 70%. Model validation in the 40 cases in clinical trial (NCT 01124253) demonstrated that the prediction sensitivity of the model was 85.7%, specificity 66.7%, Kappa: 0.618, with a high degree of consistency.

**Conclusion:**

The molecular model combining CXCR4, BSP, OPN and BMP4 could help predict the risk of bone metastasis in stage IIIa and IIIb resected NSCLC.

## Introduction

Tumor cells homing to form bone metastases is common in non-small cell lung cancer (NSCLC), just like what is seen in breast, prostate and thyroid cancers. Some patients may experience bone metastasis many years after surgery of the primary tumor. The high morbidity and significantly increased risk of fractures associated with bone metastasis seriously affect patients' quality of life. About 36% of all lung cancers and and 54.5% of stage II-IIIA NSCLC showed postoperative recurrence or metastasis [1]. Many lung cancer patients expect new and more sensitive markers to predict metastatic diseases. If bone metastasis can be predicted early enough, then effective prevention could be started and may result in an improvement in survival [2]. The molecular and cellular mechanisms leading to the development of bone metastasis in NSCLC remain unclear, therefore in this study, we investigated the current understanding of bone metastasis in NSCLC. We constructed tissue microarray, and used immunohistochemical method to assess the expression of 10 bone metastasis-related tumor markers in primary NSCLC tissue, which involved multi-step process of bone metastasis [3], including the proliferation, adhesion, escape (MMPs, OPN, c-Src) of primary tumors; targeted metastasis to bone (CXCR4); bone-specific adhesion and implantation (BSP); formation of metastases in bone (IGF1R, BMPs, PTHrP) and metastasis-associated cell signaling pathways (PI3K, NFκB). We established a molecular model composed of biological markers to predict the risk of bone metastasis in resected stage III NSCLC to screen the patients at high risk of bone metastasis for early intervention.

## Patients and methods

### Patients

The patients for establishing the model were 105 cases of pathologically-confirmed stage III NSCLC, who were the whole cohort and treated by complete resection from June 2002 to December 2006 at Shanghai Chest Hospital, and were followed up until December 2008. Before surgery, these patients did not have any chemo/radiotherapy, immunotherapy or other treatments that could significantly modulate the cancer cell biology. All the patients had complete resection of the tumor and staged accoding UICC 1999. The patients included 65 males and 40 females. The median age was 59 (34 to 76) years. Pathological examination showed 88 cases of adenocarcinoma, and 17 cases of non-adenocarcinoma. Stage IIIa was confirmed in 86 cases, and IIIb in 19 cases. Cisplatin-based adjuvant chemotherapy was administrated to patient with completely resected NSCLC. Three or more cycles of postoperative adjuvant chemotherapy were received in 76 cases. The 45 cases of bone metastasis were designated as bone metastasis group. The remaining 60 cases with visceral metastasis or without metastasis were defined as non-bone metastasis group.

The patients recruited in the validation group in the prospective model consists of 40 cases of pathologically-confirmed Stage III NSCLC the whole cohort enrolled in clinical trial (NCT 01124253), who had received complete surgical resection from July 2007 to August 2009, 26 males and 14 females. The median age was 57 (41 to 76) years. Pathological examination showed 33 cases of adenocarcinoma, and 7 cases of non-adenocarcinoma. Stage IIIa was confirmed in 35 cases, and IIIb in 5 cases.

### Preparation of tissue microarray

HE sections were examined under a microscope to identify and mark the cancer nests. HE sections were used to mark the corresponding sampling site on paraffin blocks of the donor. Preparation of tissue chip block: The ordinary pathological paraffin was melted and precipitated repeatedly for 3 times. Then 3% refined beeswax was added to prepare blank paraffin blocks of 32 mm × 15 mm × 10 mm, which were cooled at room temperature. Design and preparation of tissue microarray: an 8 × 5 tissue array was designed and made into module with the drilling system. A punch needle with a diameter of 2 mm was used to remove the tissue cores one by one from the specified site of donor paraffin blocks. The tissue cores were put into a pre-designed array module, arranged as tissue microarray. The prepared tissue microarrays were placed in an instrument at 60°C for 20 min. The modules were pressed slightly to make the tissue cores level and align in the module. The prepared module was then cut into conventional 2 μm slice and mounted on a silica slide. The slide was incubated overnight at 60°C. Each chip of the tissue microarray contained the 20 tissue samples and 2 normal controls. Each case repeated.

### Immunohistochemistry S-P assay

Mouse anti-human monoclonal antibodies against parathyroid hormone related protein (PTHrP), osteopontin (OPN), Src proto-oncogene (c-Src), matrix metalloproteinase - 2 (MMP2), chemokine receptor type 4 (CXCR4), phosphatidylinositol kinase (PI3K), bone sialoprotein (BSP), nuclear transcription factor (NFκB), insulin-like growth factor-IR (IGF-1R), bone morphogenetic protein −4 (BMP-4) were purchased from Beijing Boao Sen Biotechnology Co., Ltd. SP kit was purchased from Fuzhou Maixin Biotechnology Development Co., Ltd. Antigen retrieval was conducted according to the protocol. The known positive tissue sections of lung cancer were used as positive control, PBS instead of primary antibody as negative control. Evaluation criteria: immunostaining score was calculated based on the sum of the percent positivity of stained tumour cells and the staining intensity. The percent positivity was scored as “0” (<5%, negative), “1” (5–25%, sporadic), “2” (25–50%, focal), “3” (<50%, diffuse). The staining intensity was scored as “0” (no staining), “1” (weakly stained), “2” (moderately stained), and “3” (strongly stained). Both percent positivity of cells and staining intensity were decided under double-blind condition. The final expression score was calculated with the value of percent positivity score × staining intensity score, which ranged from 0 to 9. We defined expression level as follow: “”0 (score 0–1), “+” (score 2–3), “++” (score 4–6) and “+++” (score > 6) [4]. Expression (++) or more be considered positive.

### Follow-up and database construction

All patients were followed-up regularly by a designated staff, who collected all the information to a central database. Generally, we followed up the patients 3 to 4 times a year in the first 2 years, and once in half a year in the following 3 years. The disease control time (DCT) was defined as the time interval from surgical section to the recurrence. The last follow-up visit was on June 30, 2008. The DCT and site of recurrence were followed-up in the same way in validation group, for whom the date of last follow-up was June 30, 2011.

### Statistical analysis

SPSS 17.0 software was used for data analysis. Categorical data were described by percentage to test group differences. Logistic regression analysis was applied to estimate the parameters using maximum likelihood estimation method, α = 0.05, for establishing a model to predict the risk of bone metastasis in resected stage III NSCLC. Model fitting was evaluated by Hosmer-Lemeshow test. The model was also tested by receiver operating characteristics (ROC) analysis, and prospectively validated with kappa test. P <0.05 was considered statistically significant.

## Results

### Model group

A total of 105 cases of stage III NSCLC patients were analyzed, including 45 cases with bone metastasis, and 60 cases without bone metastasis. Only pathologic stage statistically significant difference was found between bone metastasis group and non-bone metastasis group in terms of clinical and pathological factors (Table 1).

**Table 1:** Comparison of major clinico-pathological factors between NSCLC patients with or without bone metastasis

Characteristics	Bone metastasis (n = 45)	Non-bone metastasis (n = 60)	P value
	n (%)	n (%)	
Gender			
Male	28 (62.2)	37 (61.7)	0.954
Female	17 (37.8)	23 (38.3)	
Age (mean ± SD) (yr.)	55.8 ± 12.1	57.4 ± 7.2	0.398
Histopathology			
Adenocarcinoma	39 (86.7)	50 (83.5)	0.567
Non-adenocarcinoma	6 (13.3)	10 (16.7)	
Stage			
IIIa	33 (73.3)	53 (86.7)	**0.048**
IIIb	12 (26.7)	7 (13.3)	
Adjuvant chemotherapy			
Yes	30 (66.7)	46 (76.7)	0.828
No	15 (33.3)	14 (23.3)	

### Establishment of the prediction model of bone metastasis

**Figure 1:**
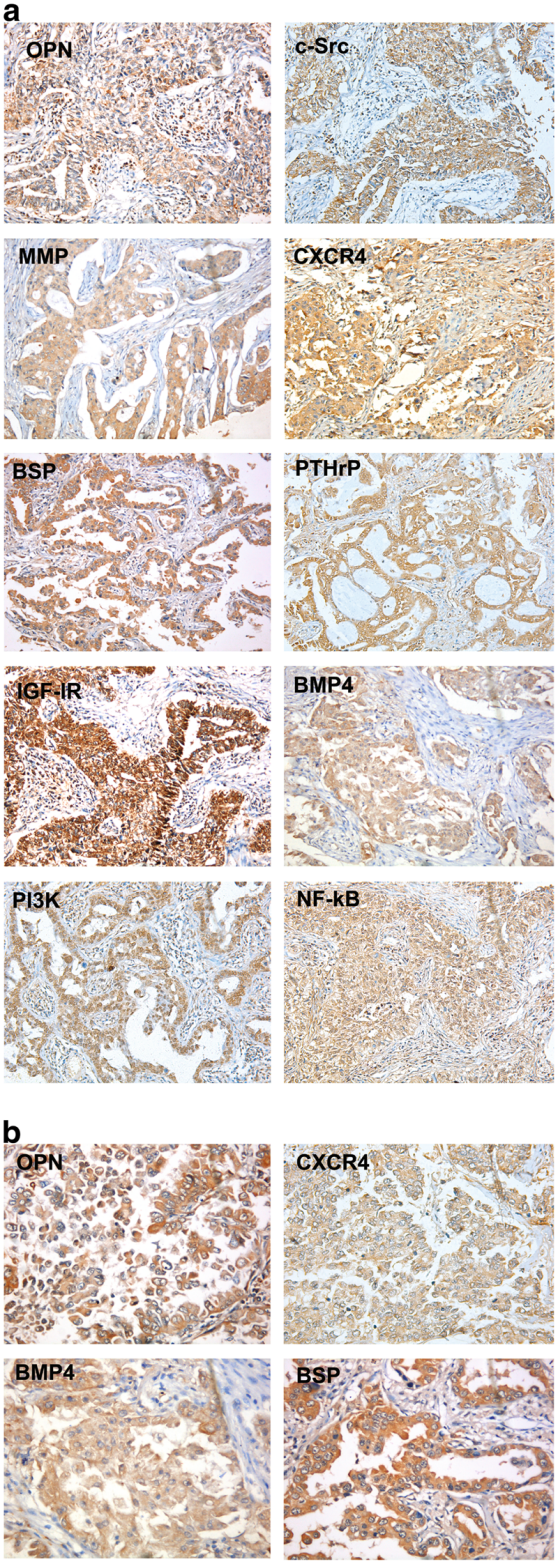
(a) Expression positive (++) of biomarkers of OPN, c-Src, MMP2, CXCR4, BSP, PTHP, IGF-1R, BMP4, PI3K and NK-kappaB (original magnification Χ100), (b) Expression of biomarkers of OPN, CXCR4, BMP4, BSP (original magnification Χ200).

**Figure 2:**
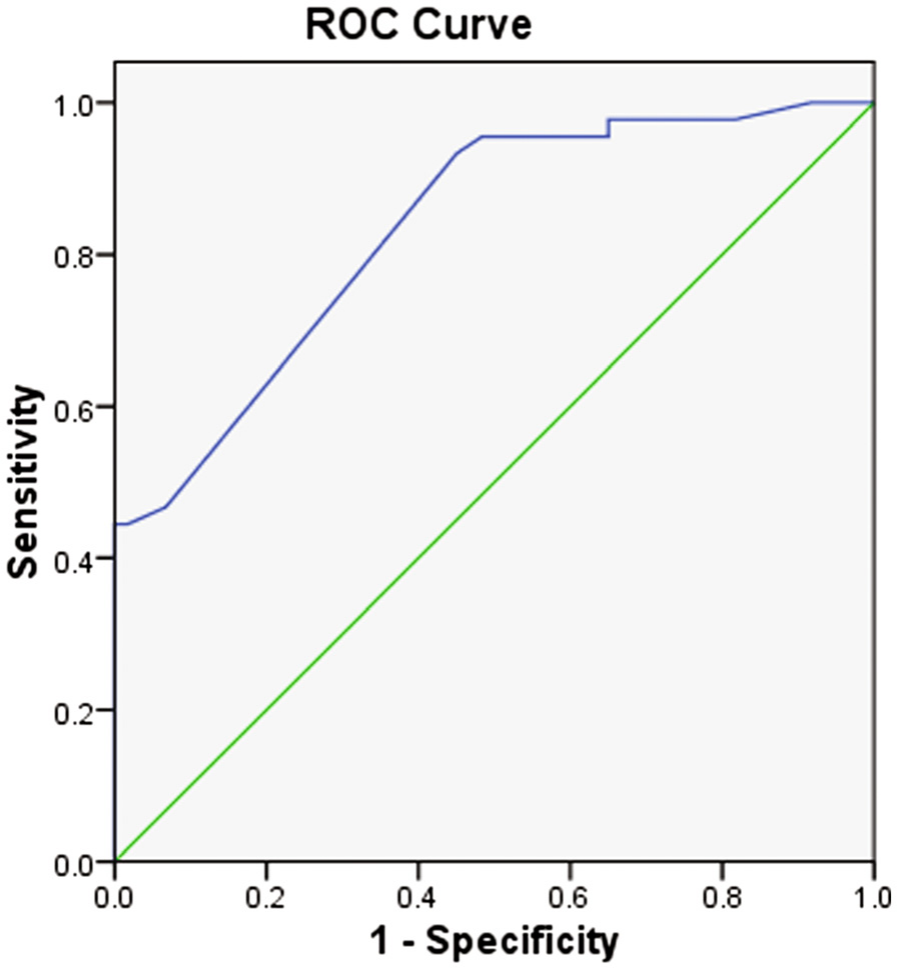
ROC curve of the biomarker model for predicting bone metastasis in resected stage III non-small cell lung cancer.

A number of cancer molecular markers associated with bone metastasis were assessed by immunohistochemical technique, including PTHrP, OPN, c-Src, MMP2, CXCR4, PI3K, BSP, NFκB, IGF-1R, and BMP4. Immunohistochemically, PTHrP, OPN, c-Src, MMP2, CXCR4, BSP, NFκB, IGF -1R, and BMP4 were mainly expressed in cytoplasm. PI3K was mainly expressed in cytoplasm, partly in the nucleus; BMP4expressed slight weakly (Figure 1). Chi-square (^2^) test showed that OPN, CXCR4, BSP, BMP4 were associated with bone metastasis (Table 2). A prediction model was established via Logistic regression analysis: logit *(P)* = − 2.538 +2.808 CXCR4 +1.629 BSP +0.846 OPN-2.939 BMP4. Hosmer and Lemeshow test *p* = 0.065. ROC test (Figure 2) suggested that the area under the curve was 81.5% (P: 0.041, 95% CI 73.4% to 89.5%). When P = 0.408, the sensitivity was up to 71%, specificity 70%. Namely, P ≥ 0.408 can be used as the screening indicator in this model to identify those at high risk of bone metastasis in resected stage III NSCLC.

**Table 2:** Correlation between cancer biomarkers and bone metastasis

Biomarkers		Bone metastasis	Non-bone metastasis	P value
		n (%)	n (%)	
**OPN**	+	40 (93.0)	48 (77.4)	**0.033**
	-	3 (7.0)	14 (22.6)	
c-Src	+	45 (100)	56 (93.3)	0.133
	-	0 (0)	4 (6.7)	
MMP2	+	38 (88.4)	47 (75.8)	0.107
	-	5 (11.6)	15 (24.2)	
**CXCR4**	+	40 (90.9)	32 (52.5)	**0.000**
	-	4 (9.1)	29 (47.5)	
**BSP**	+	44 (97.8)	49 (81.7)	**0.012**
	-	1 (2.2)	11 (18.3)	
PTHrP	+	42 (68.8)	28 (63.4)	0.647
	-	19 (31.2)	16 (36.6)	
IGF-1R	+	58 (95.1)	39 (88.6)	0.383
	-	3 (4.9)	5 (21.4)	
**BMP4**	+	22 (48.9)	51 (85)	**0.000**
	-	23 (51.1)	9 (15.0)	
PI3K	+	43 (95.6)	55 (91.7)	0.696
	-	2 (4.4)	5 (8.3)	
NFκB	+	58 (95.1)	39 (88.6)	0.383
	-	3 (4.9)	5 (21.4)	

### Prospective validation of bone metastasis prediction model

A total of 40 cases of stage III NSCLC were enrolled from July 2007 to August 2009. TMA was constructed in Dec.2010 and assessed for OPN, CXCR4, BSP and BMP4. According to this model, we predicted 8 cases would have bone metastasis and 32 cases would not. Bone metastasis was identified in 7 (17.5%) cases. Other visceral metastasis was found in 20 (50%) cases. No metastasis was identified in 13 (32.5%) cases. The prediction sensitivity of the model was 85.7%, specificity of 66.7%, Kappa: 0.618, with a high degree of consistency.

## Discussion

Bone metastases are classified as osteolylic, osteoselerotic or mixed lesions. Several molecular mechanism bring about cancer cell to metastasis to bone, and osteotropric cancer cells are believed to acquire bone cell-like properties which improve homing, adhesion, proliferation and survival in the bone microenvironment [2].

We used tissue microarray technology in this study. It is a good solution to a large volume of tumor marker tests and the comparability of results. Immunohistochemical assay was used to detect the expression of 10 molecular markers in 105 patients completely resected stage III with NSCLC tissue from the 2002 to 2006 the whole cohort. These molecular markers included PTHrP, OPN, c-Src, MMP2, CXCR4, PI3K, BSP, NFκB, IGF-1R, and BMP4. All these molecules may, individually, play important roles in breast cancer or prostate cancer bone metastasis. However, to our knowledge, there have been few studies that collectively consider all these markers and make weighted examinations of them, so as to construct a panel of makers for the prediction of NSCLC bone metastasis. Bearing this in mind, we designed this study in order to early predict the bone metastasis for more personalized targeted therapy.

Univariate analysis found that high expression of OPN, CXCR4, and BSP and low expression of BMP4 had significantly impact on bone metastasis in resected Stage III NSCLC. OPN was dominantly presented in bone matrix. It interacts with its receptor integrin vβ3 to promote cell proliferation, invasion and adhesion. Fong et al. [5] found that OPN could increase the metastasis ability of lung cancer cells through activation of integrin/FAK/AKT and NF-κB signaling pathway. In addition, OPN also increases the activity of EGFR tyrosine kinase, and Met kinase to promote tumor cell migration by inducing EGFR and HGFR (Met) expression. OPN may down-regulate the expression of Syndecan-1 to reduce the adhesion between tumor cells, and thereby encouraging tumor metastasis [6,7]. Study in metastatic breast cancer revealed [3] that the breast cancer cells high expression of CXCR4 metastasized to the corresponding target organs with high expression of CXCL12, such as lymph nodes and bone marrow. CXCR4 interacts with CXCL12 to promote tumor cell proliferation, induce the expression of MMP2, and increase invasion and metastasis. High expression of CXCR4 not only enhances distant metastasis, but also leads to more pronounced bone metastasis relative to visceral metastasis. In vitro experiments also confirmed that the high expression of CXCR4 alone was significantly associated with higher rate of bone metastasis [8]. No significant difference was found between bone metastasis group and non-bone metastasis group in this study, although MMP2 was over expressed in the patients with distant metastasis. According to the "seed and soil" theory, some tumor cells prefer to bone metastases due to their inherent biological characteristics, BSP and c-Src for example. BSP has cell adhesion function, and is involved in cell migration and signal recognition [9]. BSP acts as the ligand of integrin in osteoblasts, osteoclasts, and tumor cells of bone metastasis. It binds with integrin and play a role in osteocyte differentiation, bone matrix mineralization, as well as adhesion, proliferation and metastasis of tumor cells [10,11]. In the distant metastasis of both breast cancer and prostate cancer, the incidence of bone metastasis is higher than that of visceral metastasis in case of high expression of BSP. The level of BSP expression in the cancer cells of bone metastasis is higher than that in the primary tumor. The primary tumor with high expression of BSP may be more inclined to bone as a target organ of metastasis. And the microenvironment of bone further up-regulates the expression of the BSP, while the microenvironment of visceral organs reduces the expression of BSP. Positive expression of BSP in tumor cells suggests that BSP has contributed to bone metastasis [12]. Studies comparing bone and visceral metastasis in breast cancer indicated that up-regulation of c-Src gene can increase bone metastasis, while down-regulation of this gene will decrease the malignant phenotype of breast cancer cells, and reduce bone metastasis [13]. However, high expression of c-Src was not associated with stronger bone metastasis than other distant metastasis in this study. BMPs are bone morphogenetic proteins, which is a member of growth factor family. Functional studies have shown that BMPs are involved in both promotion and inhibition of tumor cell growth [14]. BMPs secreted by tumor cells can induce cell differentiation of osteoblasts, increase the formation of new bone and promote bone mineralization. Recent evidence suggests [15] that the BMP4-induced phenotypic changes were mediated through the activation of the canonical SMAD signaling pathway. It has been reported that BMP4 is overexpressed in melanoma cell line and lung cancer. BMP4 plays an important role in bone metastasis of prostate cancer [16], and BMP4 overexpression inhibits proliferation and induces apoptosis in many cancer cell line [15,17]. This study also showed that BMP-4 expression was lower in primary tumors. Bone metastasis of lung cancer is a dynamic process involving bone resorption resulted from tumor cell-induced osteolysis and bone formation due to osteoblasts. This study didn’t show PTHrP and IGF-1R overexpression in NSCLC tissue related NSCLC bone metastasis. PTHrP is required for colony of bone metastasis of cancer cells. It is a cytokine produced by the metastatic cancer cells [18]. But Henderson [19] had demonstrated that bone metastases that do not express PTHrP in primary breast cancer begin to do so when they reach bone. The bone microenvironment seems to provide what is needed for the breast cancer cells to produce PTHrP, even if they could not produce it before they got there. This study demonstrated that PTHrP was expressed only in 66.67% of the primary tumors. Breast cancer overexpress IGF-1R through promoting proliferation and reducing apoptosis to increase bone metastasis [20], the effects of IGF-1R have been confirmed in bone metastasis of prostate cancer [21] but the role of IGF-1R overexpress in NSCLC bone metastasis is not clear, it still needs to be further investigated.

Multivariate Logistic regression has successfully established a model for predicting the risk of bone metastasis in resected Stage III NSCLC: logit (P) = − 2.538 +2.808 CXCR4 +1.629 BSP +0.846 OPG-2.939BMP4. The area under the ROC curve was 81.5%. When P = 0.408, the sensitivity was up to 71%, specificity 70%. The model has successfully validated in 40 patients with resected stage III NSCLC from 2007 to 2009 whole cohort in clinic trial, who were followed up for 3 years. The model showed a sensitivity of 85.7% and specificity of 66.7%, Kappa: 0.618. The results are highly consistent. The model based on bone metastasis-associated biomarkers established in this study is useful in providing rationale for the screening, intervention and targeted therapy of bone metastasis in lung cancer.

Although the results are interesting, the limitations of this study should be acknowledged. The patients enrolled into the prediction model and validation model were whole cohort of completed resected stage III patients, not including patients from other groups. Therefore, there might be selection bias in the model construction and results interpretation. The results might be more suitable to clinically stage III patients. Any generalization to other stages should not be expected. In the future, a bigger study with larger sample size with different stages, could help more objectively judge the value of this prediction model.

## Competing Interests

The authors have declared that no competing interests exist.

## Authors’ contributions

ZZ carried out the protocol design, participated in the patients enrollment and TMA assay, drafted the manuscript. Z-WC carried out the patients enrollment. X-HY carried out the TMA immunohistochemistry assay. These authors contributed equally to this work. All authors read and approved the final manuscript.
